# ADARRI: a novel method to detect spurious R-peaks in the electrocardiogram for heart rate variability analysis in the intensive care unit

**DOI:** 10.1007/s10877-017-9999-9

**Published:** 2017-02-16

**Authors:** Dennis J. Rebergen, Sunil B. Nagaraj, Eric S. Rosenthal, Matt T. Bianchi, Michel J. A. M. van Putten, M. Brandon Westover

**Affiliations:** 1 Neurology Department, Massachusetts General Hospital, 55 Fruit Street, Boston, MA 02114 USA; 2 Department of Clinical Neurophysiology, Medisch Spectrum and MIRA- Institute for Biomedical Technology and Technical Medicine, University of Twente, P.O. box 217, 7500 AE, Enschede, The Netherlands

**Keywords:** Heart rate variability, ECG artifacts, Intensive care, ICU

## Abstract

We developed a simple and fully automated method for detecting artifacts in the R-R interval (RRI) time series of the ECG that is tailored to the intensive care unit (ICU) setting. From ECG recordings of 50 adult ICU-subjects we selected 60 epochs with valid R-peak detections and 60 epochs containing artifacts leading to missed or false positive R-peak detections. Next, we calculated the absolute value of the difference between two adjacent RRIs (adRRI), and obtained the empirical probability distributions of adRRI values for valid R-peaks and artifacts. From these, we calculated an optimal threshold for separating adRRI values arising from artifact versus non-artefactual data. We compared the performance of our method with the methods of Berntson and Clifford on the same data. We identified 257,458 R-peak detections, of which 235,644 (91.5%) were true detections and 21,814 (8.5%) arose from artifacts. Our method showed superior performance for detecting artifacts with sensitivity 100%, specificity 99%, precision 99%, positive likelihood ratio of 100 and negative likelihood ratio <0.001 compared to Berntson’s and Clifford’s method with a sensitivity, specificity, precision and positive and negative likelihood ratio of 99%, 78%, 82%, 4.5, 0.013 for Berntson’s method and 55%, 98%, 96%, 27.5, 0.460 for Clifford’s method, respectively. A novel algorithm using a patient-independent threshold derived from the distribution of adRRI values in ICU ECG data identifies artifacts accurately, and outperforms two other methods in common use. Furthermore, the threshold was calculated based on real data from critically ill patients and the algorithm is easy to implement.

**Electronic supplementary material:**

The online version of this article (doi:10.1007/s10877-017-9999-9) contains supplementary material, which is available to authorized users.

## Introduction

Heart rate variability (HRV) [1–5] monitoring is increasingly used in the intensive care unit (ICU) as a continuous noninvasive index with potential to serve as an early warning signal of worsening illness. Recent successful applications include detection of sepsis in neonates and adults [6–9], tracking the depth of sedation in patients on mechanical ventilation [10], and detection of delayed cerebral ischemia following subarachnoid hemorrhage (SAH) [11, 12].

Nevertheless, the presence of signal artifacts poses a challenge to routine HRV monitoring in the ICU environment. HRV measures are calculated from the R-R interval (RRI) [13] time series derived from the electrocardiogram (ECG), which requires accurate localization of QRS complexes. Failure to identify and correct for artifacts can substantially degrade the value of HRV measures.[14–17].

Practical ICU applications of HRV monitoring require fully automated ECG artifact detection methods. The method in widest use was proposed by Berntson et al. [18], and is based on the distribution of differences in RRIs. Because RRI differences arising from artifacts are typically large compared to valid RRIs, large RRI differences serve to identify potential artifacts. However, this algorithm relies on statistics derived from the RRI time series of an individual’s ECG, and is thus patient dependent, i.e. will behave differently in different patients, particularly in high-artifact settings. Moreover, the method was validated on data from chimpanzees and young healthy volunteers with simulated artifacts. Thus it is unclear how effectively this algorithm may perform in an actual ICU setting. Clifford et al. [14] developed a simpler, patient-independent automatic artifact identification algorithm based on variation of adjacent RRIs. Their method exhibited a moderately good accuracy of 67%, but also was not validated in critically ill patients or in the ICU setting.

To overcome these limitations, we developed a simple and fully automated method for detecting artifacts in the RRI time series that is tailored to ICU data. Our intended application is monitoring HRV in patients with subarachnoid hemorrhage.[11, 12] Thus, we optimized the algorithm parameters and validated its performance on a large set of ICU ECG recordings from a diverse group of critically ill patients with SAH. We present our method and compare the performance with the Berntson and Clifford methods on the same data.

## Methods

### Patient selection

Archived ECG recordings were retrieved for analysis from patients admitted to the Massachusetts General Hospital (MGH) Neurosciences Intensive Care Unit (NICU). We selected convenience samples of 50 subjects admitted between 2012 and 2015 for whom archived ECG recordings were available. This study was carried out under a protocol approved by the local institutional review board (IRB). Patient consent was not required.

### Data acquisition

ECGs were recorded at 240 Hz. Training data for algorithm development was established as follows. From each patient’s ECG, 60 s-long epochs with only valid R-peak detections and 60 s-long epochs containing artifacts were selected visually as described below by one of the authors with clinical expertise (MBW). Putative R-peaks were automatically detected within these 60 s segments using the Pan-Tompkins algorithm [19]. A valid epoch was defined as one in which every detected R-peak corresponded to a real R-peak, and in which no R-peak was missed. All other epochs were defined as “artifact” epochs. Note that artifact epochs typically contained at least some valid R-peaks in addition to missed peaks and false detections. All analysis was performed in Matlab (R2014b, Mathworks, Natick, MA, USA).

### RRI and adRRI

ECG artifacts cause errors in the RRI interval time series either via missed R-peaks or false detected R-peaks. Both of these error types result in larger-than-typical absolute differences between adjacent RRIs. Therefore, large jumps in the difference between consecutive RRI values have an increased probability of being due to an artifact compared with differences arising from variation in valid RRIs. Our approach attempts to identify a single optimal threshold value for the absolute difference of the RRI by which to discriminate valid R-peaks from artifacts. We postulated that a single optimal threshold can be obtained that can satisfactorily detect artifacts across ECGs from different patients.

As candidate features for artifact detection, we examined both the RRI and the absolute value of the difference between two adjacent RRIs: the adRRI. The RRI is defined as:

1 $$RR{{I}_{n}}={{R}_{n+1}}-~{{R}_{n}}$$

with *R*_*n*_ as the *n*th detected R-peak. The RRI is the time between two adjacent detected R-peaks: *R*_*n*_ and *R*_*n*+*1*_ (for which R is a detected R-peak by the Pan-Tompkins algorithm [19]). The adRRI is defined as:

2 $$adRR{{I}_{n}}=~\left| RR{{I}_{n}}-RR{{I}_{n+1}} \right|$$

and is the absolute value between two adjacent RRIs: *RRI*_*n*_ and *RRI*_*n*+*1*_. Because values of RRIs and adRRIs span a wide range, for the purposes of plotting distributions of these values hereafter in plots we will work with their logarithms to allow better visualization.

**Fig. 1:**
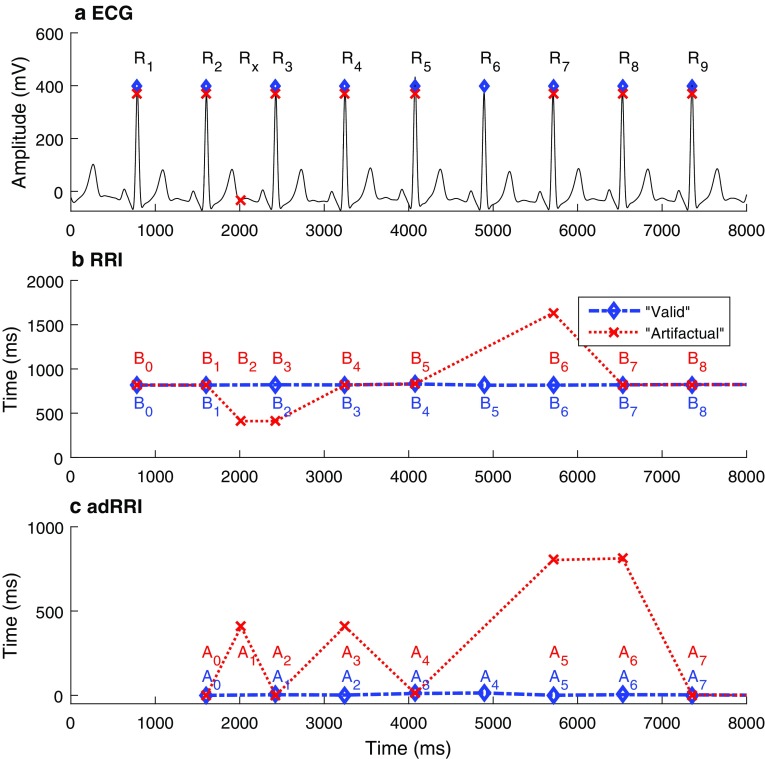
Overview of the RRI and adRRI derived from R-peak detection of the ECG. In **a** the ECG is shown where the blue signal represents a series of completely valid peak detections and the red signal represents a series of peak detections containing two errors (resulting from application of the Pan-Tompkins algorithm [19]). The two errors include a spurious peak (*R*_*x*_) and a missed peak (*R*_*6*_). In **b** the RRI is shown as *B*_*n*_, calculated as $Bn~ = ~{R_{n + 1}} - {R_n}$. The valid peak detections (*blue*) correspond to an essentially constant sequence of RRIs. For the red detections, the spurious R-peak *R*_*x*_ results in two decreased RRIs (*red B*_*2*_, *B*_*3*_) and a missed peak *R*_*6*_ results in an increased RRI value (*red B*_*6*_). In **c** the adRRI is shown as *A*_*n*_, calculated as ${A_n} = ~\left| {{B_n} - {B_{n + 1}}} \right|$. The valid peak detection (*blue*) has a constant value around zero, but for the red signal both a spurious detected (*A*_*1*_, *A*_*3*_) peak and a missed R-peak (*A*_*6*_, *A*_*7*_) result in an increased adRRI, signaling the presence of an artifact

As noted above, there are two possible types of artifacts: either a spurious R-peak is falsely detected, or a real R-peak is missed. In general, a spuriously detected peak results in a shorter RRI, while a missed peak results in a longer RRI. This requires two thresholds for RRI artifact detection: a low RRI threshold for spurious peaks and a high RRI threshold for missed peaks. For the adRRI, both spurious and missed peaks result in an increased absolute difference between RRIs compared with those arising from valid R-peaks. For this reason, only a single threshold is required for artifact detection with the adRRI. This is illustrated in Fig. 1.

### Definition of true labels

We developed two different versions of our algorithm (two different threshold values), based on (1) coarse-grained labeling of entire epochs as either artefactual versus valid, and (2) fine-grained labeling of individuals R-peaks as being artefactual versus valid.

#### Labeling of epochs

To allow detection of epochs containing artifacts, one of the authors (MBW) visually inspected all epochs and labeled each as “valid”, containing no artefactual R-peak detections; or “artifact”, containing one or more artefactual R-peak detection. Note that “artifact” epochs may contain a mixture of correct and spurious R-peak detections

#### Labeling of individual R-peaks

**Fig. 2:**
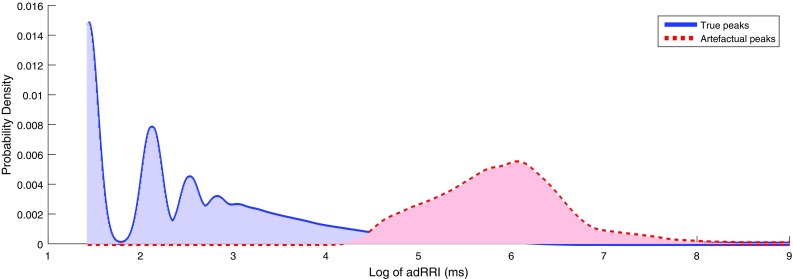
Kernel probability density function estimation of the adRRI. The log of the distribution of the individual true peaks (*blue*) and artifact peaks (*red*) of the adRRI estimations for all subjects. The adRRI of the true peaks are much lower than the adRRI of artifact peaks, although there is a small overlap

We labeled individual peaks in artifact epochs by a three-step series of manual and computational methods guided by direct visual inspection, as follows. First, for each case we calculated individualized thresholds, in two ways: to maximize the accuracy in discriminating between either the log-RRI or the log-adRRI values from the manually labeled “valid” and “artifact” within that ECG (see Fig. 2). Second, using these thresholds (two for the log-RRIs, one for the adRRI), we performed an initial categorization of individual R-peaks within artifact intervals as true vs artefactual detections. Finally, we added the false flag alarm (FA) of Berntson et al. [18] to further improve the accuracy of the peak detections (see supplement 1 for further details).

Empirically, we found that the three-step procedure described above generally gave better performance when relying on log-adRRI than on RRI. Accordingly, the final labels for individual artifact peaks were defined using the above multi-step procedure using the log-adRRI labels.

### Artifact identification methods

We compared the performance of three methods on our ICU ECG data, hereafter designated as (A), our new method: the ‘ADARRI’ (Absolute value of the Difference between Adjacent RR Intervals) method; (B), Berntson’s method, and (C) Clifford’s method. Whereas both method A and B use the absolute difference between two RRIs to detect artifacts, method C applies the relative difference between two adjacent RRIs. We hypothesized that combining these methods might improve accuracy for artifact identification. Hence, we also evaluated sequential application of a combination of methods A and C (AC), and methods B and C (BC). Finally, we evaluated whether applying the FA detection routine of Bertnson et al. [18] (hereafter referred to as “FA-edit”) was able to improve the performance of method A. The FA-edit routine attempts to refine or “edit” the initial artifact detections by identifying detections that are likely to be incorrect (false positives). For combined methods AC and BC, a detected R-peak was labeled as an artifact if method 1 OR/AND method 2 identifies it as artifact. This resulted in six different artifact detection methods: (1) A; (2) A + FA-edit; (3) B; (4) C; (5) AC; (6) BC. The working mechanism of the ADARRI method (A), Berntson’s method (B) and Clifford’s method (C) are explained next.

#### ADARRI method (A)

 Our artifact identification algorithm is based on a single threshold value, derived from the log-adRRI distributions of artifacts and valid data pooled from all 50 patients. This threshold was calculated within the range of [1–500] (with step size = 1) as the threshold with the highest accuracy (accuracy = (true positive + true negative)/total detections) for artifact detection, based on balanced sets of epochs with valid R-peaks and artifacts from all 50 subjects (see statistical analysis below regarding data balancing).

#### Berntson method (B)

Berntson’s method is also based on differences between the distributions of valid and abnormal R-peak detections. The threshold in method B is derived from the interquartile range and the median of the probability distribution of adRRI values. Berntson’s method includes also an algorithm to identify and remove false alarms (FA, i.e. falsely-detected artifacts), using the same threshold for artifact detection (see supplement 1 for more detail). For method B patient individual thresholds were calculated based on the interquartile range and the median of the probability of all the adRRI values for the subject.

#### Clifford method (C) 

For the Clifford method, an R-peak was labeled as an artifact if the RRI differs by more than 20% from the previous RRI.

We evaluated the overall performance on all 50 patients for all six methods described above: A, A + FA-edit, B, C, AC, BC based on balanced samples of valid R-peaks and artifacts from all 50 subjects. Furthermore, we evaluated the performance of methods A, B and C for all 50 patients individually. These methods are tested in two ways: (1) for the detection of “*artifact containing epochs*”, which labels the whole epoch as “artifact” if an artifact was detected and (2) “artefactual R-peak”, which labeled each *individual R-peaks* as “valid” or “artifact”. These are further denoted as *epoch evaluation* and *individual R-peak evaluation*, respectively.

### Statistical analysis

For each method we calculated the sensitivity (SE), specificity (SP), positive predictive value (PPV) and both the positive and negative likelihood ratio (LR+ and LR−) for detecting artifacts and are defined as:

3 $$LR+~=~\frac{Sensitivity}{1-Specificity}$$

4 $$LR-~=~\frac{1-~Sensitivity}{Specificity}$$

Performance measures were plotted in receiver operating curves (ROC) and precision-recall (PR) curves. For method A and A + FA-edit the area under the curve (AUC) was calculated. For individual patient performance, the SE, SP and PPV, LR+ and LR− were calculated as medians (interquartile range) over all 50 subjects.

To deal with the imbalanced number of valid and artefactual peaks, we generated balanced sets by choosing randomly without replacement from the larger group to yield groups of equal size. All performance statistics were then calculated on the balanced dataset. This balancing procedure was repeated 20 times and the average values were used as the final reported performance measures.

## Results

### Patient selection

Five “artifact” epochs were excluded due to a complete absence of data within the epoch (hence no R-peak detections), yielding 2995 “valid” and 3000 “artifact” epochs. Among 257,738 total R-peak detections, 235,644 (91.5%) were true detections and 22,094 (8.5%) were due to artifacts. The mean heart rate for valid epochs was 79 ± 16 bpm and for “artifact” epochs 78 ± 23 bpm.

### Overall performance

**Fig. 3:**
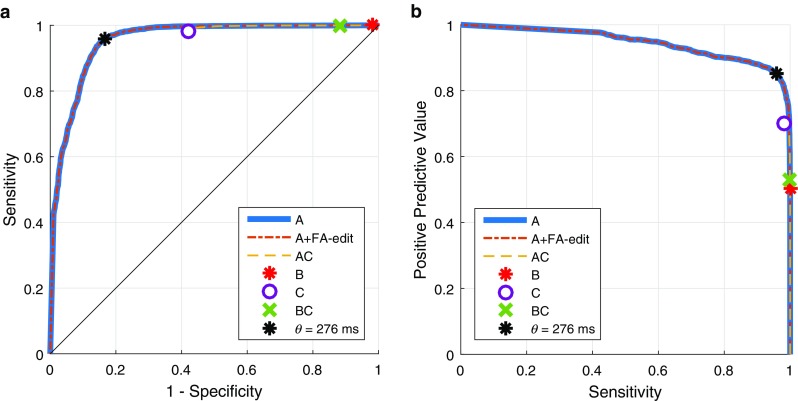
Overall performance of epochs. A = ADARRI method, A + FA-edit = Method A with FA-edit, B = Berntson method, C = Clifford method, AC = combination of A and C and BC = combination of B and C. **ӿ** = Optimized threshold value for method A. **a** ROC curves of overall performance of epochs. Method A and A + FA-edit have the highest accuracy with SE = 96% and SP = 83%. **b** The PR curves of overall performance of epochs. Method A and A + FA-edit show the best performance with SE = 96% and PPV = 85%

**Fig. 4:**
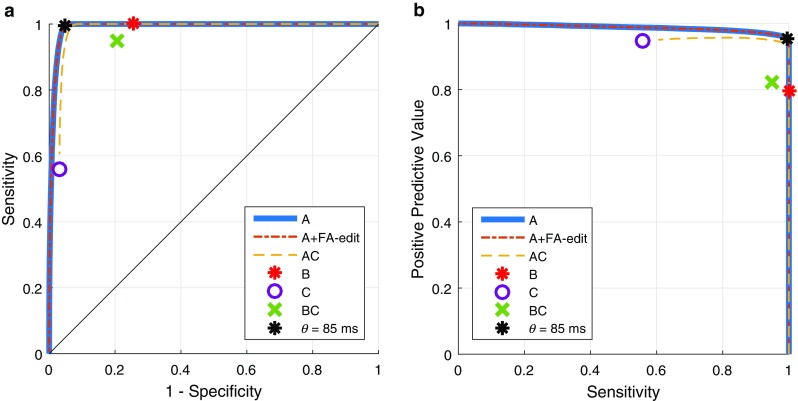
Overall performance of individual R-peaks. A = ADARRI method, A + FA-edit = Method A with FA-edit, B = Berntson method, C = Clifford method, AC = combination of A and C and BC = combination of B and C. **ӿ** = Optimized threshold value for method A. **a** The ROC curves of overall performance of individual R-peaks. Method A, A + FA-edit and AC have the highest accuracy with SE = 99% and SP = 95–92%. **b** The PR curves of overall performance of individual R-peaks. Method A, A + FA-edit and AC have the best performance with SE = 99% and PPV = 95–93%

Overall performance results for the six different methods (A, A + FA-edit, B, C, AC, BC) are shown in ROC and PR plots in Figs. 3 and 4 for epochs and individual R-peaks, respectively. The optimal overall threshold for method A was θ = 276 ms for detecting artifact-containing epochs and θ = 85 ms for detecting individual abnormal R-peaks; the mean patient-optimized threshold for method B was θ = 18 (10–40) ms.

Inspecting the ROC and PR curve for *epoch evaluation* (Fig. 3), the curves for method A, A + FA-edit and AC span a wide range of possible performance set points depending on the chosen threshold value. Methods B, C and BC exhibit only a single operating point, because they each use a fixed threshold value. Method A and A + FA-edit show identical performances (AUC = 95.26). Method AC overlaps partially with method A and A + FA-edit, but cannot achieve points with SP higher than 58%. Method A (as well as A + FA-edit) performs best since its ROC curve is above all the other methods. At the threshold value that provides maximum accuracy (θ = 276 ms), method A obtains SE = 96%, SP = 83%, PPV = 85%, LR + = 5.9 and LR−= 0.056.

Inspecting the ROC and PR curve for *individual R-peak evaluation* (Fig. 4), again the curves for method A, A + FA-edit and AC exhibit a wide range of possible performance values as a function of the chosen threshold value. Also here, method B, C and BC use a fixed threshold which results in single operating points. Method A (AUC = 99.07%) performs negligibly better than A + FA-edit (AUC = 99.06%). Method AC partially overlaps with method A and A + FA-edit, but cannot achieve SP values below 97%. Method A performs best overall. At the threshold value that provides maximum accuracy (θ = 85 ms) method A obtains SE = 99%, SP = 95%, PPV = 95%, LR + = 21.9 and LR− =  0.08. The overall performance statistics for all methods are shown in Table 1.

**Table 1:** Performance of all methods based on all subjects

	SE (%)	SP (%)	PPV (%)	LR+	LR−
Epoch evaluation					
Method A	96	83	85	5,6	0,048
Method B	100	2	50	1,0	0,000
Method C	98	58	69	2,3	0,034
Method AC	98	58	69	2,3	0,034
Method BC	100	12	53	1,1	0,000
Individual R-peak evaluation					
Method A	99	95	95	19,8	0,011
Method B	100	74	79	3,8	0,000
Method C	56	97	95	18,7	0,454
Method AC	99	92	93	12,4	0,011
Method BC	95	79	82	4,5	0,063

Sensitivity (SE), specificity (SP), precision (PPV) and positive and negative likelood ratio (LR+, LR−), displayed as percentages of the total RRIs of all subjects. Method A uses a threshold of θ = 276 ms for epoch evaluation and θ = 85 ms for individual R-peak evaluation. Method B uses patient-optimized thresholds. Method AC shows the same results as method C for epochs and method A for individual R-peaks. Method A + FA-edit is not displayed because it has the same performances as method A

### Individual patient performance

Inspecting performance of the methods optimized for individual patient performance, in the case of *epoch evaluation*, method A performs best with SE = 100%, SP = 75%, PPV = 80%, LR + = 4, LR− = 0. Method B shows also a high SE of 100% and low LR− = 0, but has low SP (2%), PPV (50%), LR + = 1. Method C performs moderately well with a SE = 90%, SP = 60% PPV = 68%, LR + = 2.3 and LR−  = 0.167.

For *individual R-peak evaluation*, method A, optimized for best performance across aggregated patient data, achieves excellent performance (SE = 100%, SP = 99%, PPV = 99%, LR + = 100, LR− = 0); this near-perfect performance is expected due to way the labels were defined (see Sect. “methods”) Using individually-optimized thresholds, method B performs with a high SE of 99% and LR− of 0.013, but with a moderate SP of 75%, PPV 82% and LR+ of 4.5. Method C performs with high SP of 98%, PPV 96% and LR+ of 27.5, but has a low SE of 55% and LR− of 0.46. The results of method A, B and C in order to optimize performance in individual patient ECG recordings are showed in Table 2.

**Table 2:** Performance of method A, B and C for individual subjects

	SE (%)	SP (%)	PPV (%)	LR+	LR−
Epoch evaluation					
Method A	100 (100–100)	75 (41–90)	80 (63–91)	4 (1.8–10)	0 (0–0)
Method B	100 (100–100)	2 (0–8)	50 (50–52)	1.0 (1.0–1.1)	0 (0–0)
Method C	90 (85–92)	60 (35–78)	68 (58–80)	2.3 (1.3–4.6)	0.167 (0.429−0.103)
Individual R-peak evaluation					
Method A	100 (100–100)	99 (96–100)	99 (96–100)	100 (25–inf)	0 (0–0)
Method B	99 (99–100)	78 (72–92)	82 (78–93)	4.5 (3.5–14.3)	0.013 (0.014–0)
Method C	55 (52–59)	98 (96–99)	96 (93–97)	27.5 (13–59)	0.460 (0.5–0.414)

The performance of individual subject artifact classification according to method A (an overall optimized threshold), method B (patient-optimized threshold value) and method C, which does not use a threshold. The SE, SP, PPV, LR+ and LR− are displayed as medians and interquartile ranges (25–75%) of all 50 subjects

## Discussion

We present the novel ADARRI algorithm to detect artifacts in RRI time series derived from ECG recordings in ICU patients. The method exhibits high accuracy for evaluation of epochs with a performance of SE = 100%, SP = 75%, PPV = 80%, LR + = 4, LR− = 0 and even higher performance for evaluation of individual R-peaks, with an overall performance of SE = 100%, SP = 99%, PPV = 99%, LR + = 100 and LR− = 0. On our data, the new method outperforms two other commonly used methods.

Berntson et al’s [18] method achieved SE of 100% and SP of 99% on healthy subjects with added simulated artifacts. In our ICU population, we found a SE of 99% and SP of 78% for individual peaks when using the individually optimized thresholds of Berntson. On the same ICU data, our method performed better with a SE of 100% and SP of 99%, suggesting that our method with a single threshold identifies artifacts more reliably than Berntson’s method with individualized threshold values.

Furthermore, the threshold required in Berntson’s method depend on estimates of the interquartile range and the median of the probability distribution of adRRI values for data from each patient’s data, and thus implicitly relies on the majority of the data being free of artifacts to ensure consistent results. This assumption may not hold in the ICU setting where artifacts are abundant. Kaufmann et al. [20] developed an HRV analysis program and implemented the Berntson’s algorithm to detect artifacts in the RRI time series [18]. They argued that specific threshold settings are not applicable across patients because of differences in mean RRIs between patients. However, we found that our method using a globally optimized threshold achieved a better sensitivity, specificity, positive predictive value and positive and negative likelihood ratios than Berntson’s method with individualized thresholds.

Clifford et al. [14] investigated the relation of ectopic beats and artifacts with the time of the day, heart rate and state changes. Their ECG data also came from healthy subjects and resulted in a sensitivity of 67%. Using Clifford’s method on our data, we found a sensitivity of 56% with corresponding specificity and positive prediction values >95% for R-peak evaluation. In contrast, our new method obtained a sensitivity of 99% and SP and PPV both of 99%. These results suggest that Clifford’s method does not have sufficiently high accuracy in critically ill patients as we found using our method.

Inspection of the ROC and PR curves for individual R-peaks shows that a combination of Berntson’s and Clifford’s methods improves the performance over method B or C alone. However, Berntson’s method as well as Clifford’s method combined with our ADARRI method do not obtain better performance than our method alone. Furthermore, FA-edit does not improve the performance of our method.

The primary strength of our study is that we used continuous ECG recordings of critically ill patients instead of healthy subjects. In addition, the large number of epochs and individual peaks we used argues for generalizability of our results. Finally, we conducted a thorough comparison with two existing methods in common use, thus providing evidence for the advantages of the new method presented herein.

It was not possible to visually inspect and confirm all 257,458 R-peaks to confirm that the correctness of the labels of “true” and “artifact” assigned by our three-step data labeling method. Rather, while we manually identified epochs containing artifacts, the subsequent steps used to label individual peaks within each epoch as valid versus artefactual were automated. It is thus possible that an expert might disagree with some of the resulting label assignments. However, based on the design of our method and visual inspection of selected samples, we are confident that the fraction of mislabeled peaks is negligibly small.

An important limitation of our work is that we do not investigate the *cause* of abnormal R peak detections. While we have described our task as detecting “artifacts”, in some cases beats that are statistical outliers may have important physiological implications. Examples include long pauses or “dropped beats”, and episodes of extreme tachycardia (e.g. atrial flutter or ventricular tachycardia). An important future direction for our work is thus to explore ways to discriminate between abnormal beats that represent true artifact (noise) vs those which represent clinically significant events. Nevertheless, it is still useful to identify and remove highly abnormal beats regardless of their source for applications that depend on measures of HRV. This is because HRV applications are primarily concerned with extracting information from the ECG about the state of the autonomic nervous system, whereas ectopic beats arise from processes within the heart itself [15].

A second limitation is that, similar to other work, our approach relies on visual analysis by a clinical expert to define the gold standard, i.e. which detected ECG beats are artefactual. While we feel that this approach is justified (the majority of ECG artifacts are obvious on visual inspection), an even more rigorous approach would be to evaluate the algorithm against an artifact-free dataset—a “platinum standard”. This might be possible in future work, for example, by making ECG recordings in a cohort of ICU patients using 12-lead ECGs or invasive recordings (e.g. in patients with pacemakers), and using advanced methods or visual review by multiple cardiologists to label all beats as true versus artefactual.

HRV monitoring is a continuous, noninvasive tool increasingly used in the ICU in order to serve as a warning system and early detection application for worsening illness in critically ill patients [1]−[5] Recent applications of HRV monitoring include early diagnosis of sepsis in neonates [6, 8, 9] and adults [7], monitoring the depth of sedation in mechanically ventilated ICU patients [10] and preclinical detection of major adverse cardiopulmonary events [12] as well as secondary complications [11] after SAH. As these applications become crucial in the ICU setting, artifact-free HRV monitoring will be imperative. Furthermore, correct localization of the QRS complexes in order to obtain the HRV is a challenging task in the presence of noise and artifacts in the ICU environment [13]. Failure to identify and correct for artifacts can substantially degrade the value of HRV measures.[14]–[17] Our method provides a tool for substantially improving the quality of HRV analysis by accurately identifying artefactual R-peak detections.

## Conclusion

In this study, a novel “ADARRI” algorithm based on the absolute value of differences between adjacent RR Intervals (adRRI) was introduced to identify artefactual R-peaks in the ECG for HRV analysis. This method employs a single, patient-independent threshold derived from the differences between the adRRI distributions for valid vs artefactual detections. Compared to two other methods in common use, and variations on these methods, ADARRI identifies artifacts more accurately. Furthermore, our algorithm was derived from real data obtained from ECG recordings of critically ill patients in a noisy hospital environment, the ICU. This simple algorithm provides a useful preprocessing step for analyses of HRV in the ICU setting.

## Electronic supplementary material

Below is the link to the electronic supplementary material.

Supplementary material 1 (DOCX 171 KB)

## References

[1] Sztajzel J (2004). “Heart rate variability: A noninvasive electrocardiographic method to measure the autonomic nervous system”. Swiss Med Wkly.

[2] T. F. of T. E. S. of Cardiology and T. N. A. S. of P. and Electrophysiology (1996). “Heart rate variability: Standard of measurement, physiological interpretation, and clinical use”. Eur Heart J.

[3] Bouziane A, Yagoubi B, Vergara L, Salazar A, Box PO. “The ANS sympathovagal balance using a hybrid method based on the wavelet packet and the KS-segmentation algorithm,” In: Mastorakis NE and Bojkovic Z, editors. Mostaganem, Valencia, pp.75–83.

[4] Park S, Kaffashi F, Loparo Ka, Jacono FJ (2013). “The use of heart rate variability for the early detection of treatable complications after aneurysmal subarachnoid hemorrhage”. J Clin Monit Comput.

[5] Reyes del Paso Ga, Langewitz W, Mulder LJM, van Roon A, Duschek S (2013). “The utility of low frequency heart rate variability as an index of sympathetic cardiac tone: a review with emphasis on a reanalysis of previous studies”. Psychophysiology.

[6] Griffin MP, O’Shea TM, Bissonette EA, Harrell FE, Lake DE, Moorman JR (2003). “Abnormal heart rate characteristics preceding neonatal sepsis and sepsis-like illness”. Pediatr Res.

[7] Ahmad S, Ramsay T, Huebsch L, Flanagan S, McDiarmid S, Batkin I, McIntyre L, Sundaresan SR, Maziak DE, Shamji FM, Hebert P, Fergusson D, Tinmouth A, Seely AJE (2009). “Continuous multi-parameter heart rate variability analysis heralds onset of sepsis in adults”. PLoS ONE.

[8] Lake DE, Richman JS, Griffin MP, Moorman JR (2002). “Sample entropy analysis of neonatal heart rate variability”. Am J Physiol Regul Integr Comp Physiol.

[9] Moorman JR, Carlo WA, Kattwinkel J, Schelonka RL, Porcelli PJ, Navarrete CT (2011). “Mortality reduction by heart rate characteristic monitoring in very low birth weight neonates: a randomized trial”. Natl Institutes Heal.

[10] Nagaraj SB, McClain LM, Zhou DW, Biswal S, Rosenthal ES, Purdon PL, Westover MB (2016). “Automatic Classification of sedation levels in ICU patients using heart rate variability,”. Crit Care Med.

[11] Schmidt JM, Sow D, Crimmins M, Albers D, Agarwal S, Claassen J, Connolly ES, Elkind MSV, Hripcsak G, Mayer Sa (2014). “Heart rate variability for preclinical detection of secondary complications after subarachnoid hemorrhage”. Neurocrit Care.

[12] Schmidt JM, Crimmins M, Lantigua H, Fernandez A, Zammit C, Falo C, Agarwal S, Claassen J, Mayer SA (2014). “Prolonged elevated heart rate is a risk factor for adverse cardiac events and poor outcome after subarachnoid hemorrhage”. Neurocrit Care.

[13] Antink H, Brüser C, Leonhardt S (2015). Detection of heart beats in multimodal data: a robust beat-to-beat interval estimation approach. Physiol Meas..

[14] Clifford GD, McSharry PE, Tarassenko L (2002). “Characterizing artefact in the normal human 24-hour RR time series to aid identification and artificial replication of circadian variations in human beat to beat heart rate using a simple threshold”. Comput Cardiol.

[15] Clifford GD (2002). “Signal processing methods for heart rate variability”.

[16] Kamath MV, Watanabe MA, Upton ARM (2013). Heart rate variability (HRV) signal analysis; clinical applications.

[17] Berntson GG, Stowell JR (1998). “ECG artifacts and heart period variability: don’t miss a beat!”. Psychophysiology.

[18] Berntson GG, Quigley KS, Jang JF, Boysen ST (1990). “An approach to artifact identification: application to heart period data.”. Psychophysiology.

[19] Hamilton PS, Tompkins WJ (1986). Quantitative investigation of QRS detection rules using the MIT/BIH arrhythmia database. IEEE Trans Bio-Med Eng.

[20] Kaufmann T, Sütterlin S, Schulz SM, Vögele C (2011). “ARTiiFACT: a tool for heart rate artifact processing and heart rate variability analysis”. Behav Res Methods.

