# Supplementary material for: ADARRI: a novel method to detect spurious R-peaks in the electrocardiogram for heart rate variability analysis in the intensive care unit
Source: J Clin Monit Comput. 2017 Feb 16;32(1):53–61. doi: 10.1007/s10877-017-9999-9 (PMC5559344; doi:10.1007/s10877-017-9999-9)
Supplement: Supplementary file 1 — Supplementary material 1 (DOCX 171 KB) [file 10877_2017_9999_MOESM1_ESM.docx]

# Supplemental Material

## Supplement 1: False alarm detection algorithm of Berntson et al.[18]

A spuriously detected R-peak results in 2 artefactual beats. If these 2 artefactual beats are added up and the new obtained adRRI is below threshold (θ), the actual beat is regained without loss of information. Similarly, if an R-peak was missed, this beat should be the sum of 2 valid beats. Although the precise location of the beat is lost, dividing the artefactual beat in half would result in 2 beats close to the original periods. Again, the new obtained adRRIs should be below threshold. Flowcharts of this algorithm are shown in Supplement Figures S1-S3.

The first step is the START program, which checks if the adRRI is below threshold value θ (see Figure Supplement 1). B_n_ corresponds to the evaluated RRI. The absolute difference between B_n_ and the next beat is noted as |B_n_ – B_n+1_| and corresponds to the adRRI. The prior adRRI should be below threshold value θ, otherwise the artifact cannot be evaluated. If the prior adRRI passes the threshold value, the algorithm identifies whether the beat is too long or too short by comparing the length of the beat with the next beat. If the artifact was a missed beat, the control passes to the LONG BEAT flowchart (Supplement Figure 2). If the artifact was a spurious beat, the control passes to the SHORT BEAT flowchart (Supplement Figure 3).

If the beat is considered a long beat, the algorithm continues to the LONG BEAT flowchart (Supplement Figure 2) and the beat is divided in half. The resulting adRRIs are tested against the threshold value with the surrounding beats. If these new adRRIs are below the threshold value, the FA flag is turned on and the artifact flag is turned off, resulting in recovery of the detected artifact.

If the beat is considered a short beat, the algorithm continues to the SHORT BEAT flowchart (Supplement Figure 3). To restore the spurious beat, the next beat should be a short one too. Next, the beat is summed up with the shortest surrounding beat, which results in new adRRIs. If these new adRRIs are below threshold value, the FA flag is turned on and the artifact flag is turned off, resulting in recovery of the detected artifact.

# Supplementary Figures

## Fig S1 Flowchart of the start program for artifact identification

B_n_ represent the RRI and |B_n_ –B_(n+1)_| is considered the adRRI. If the adRRI is below the threshold value, the artifact flag turns on. Next, if the difference between the RRI is and the previous RRI is below the threshold value, the false alarm flowchart continuous, otherwise the beat is not evaluated.

## Fig S2 Flowchart of the long beat program for artifact identification

### B_2_ is consist of 2 actual beats and results in a prolonged RRI and increased adRRI. If the adRRI between B_3_ and B_4_ is below threshold value again, the flowchart continuous and the “missed beat” is placed in the middle of B_2_. If this new beat results in adRRIs below threshold value, the artifact flag is turned off and the missed beat is recovered.

## Fig S3 Flowchart of the short beat program for artifact identification

### B_2_ is considered a spurious beat and results in a shortened RRI and increased adRRI. If the adRRI between B_3_ and B_4_ is below threshold value again, the flowchart continuous and the “spurious beat” of the shortest RRI is removed. If this new beat results in adRRIs below threshold value, the artifact flag is turned off and the spurious beat is recovered.
